# Burr Hole Hematoma Evacuation of Large Subdural Component Using Recombinant Tissue-Type Plasminogen Activator and a Novel Catheter: Case Report

**DOI:** 10.7759/cureus.24242

**Published:** 2022-04-18

**Authors:** Bernardo A Monaco, Evan Krueger, Sauson Soldozy, Jonathan R Jagid, Joacir G Cordeiro

**Affiliations:** 1 Neurological Surgery, University of Miami, Miami, USA; 2 Neurological Surgery, University of Sao Paulo, Sao Paulo, BRA

**Keywords:** case report, chronic subdural hematoma, subdural drainage, neurocritical care, acute subdural hematoma

## Abstract

The large acute component in a chronic subdural hematoma (cSDH) typically requires a craniotomy. Open surgery can be associated with increased morbidity and is not always possible due to systemic conditions. We present the case of a 58-year-old patient who presented with a Glasgow Coma Scale (GCS) of three fixed pupils, but remaining brainstem reflexes were present. Brain CT showed a large mixed subdural left chronic hematoma, with a predominant acute component, with a 26mm midline shift. The patient was hemodynamically unstable and coagulopathic; thus, emergency bedside burr hole evacuation was done. An “anti-thrombotic catheter” was left in the subdural space as a postoperative drain. Postoperatively, GCS improved, and CT presented a residual 12.7mm midline shift due to the acute bleeding component. Recombinant tissue-type plasminogen activator (r-tPA) solution was repeatedly administered using the catheter for two days, and it continued to drain for 10 more days with no additional dose. The patient presented clinical and radiological improvement with the dissolution of the acute component. This case is the first description of local subdural use of r-tPA to treat the acute component of cSDH with success associated with an anti-thrombotic catheter.

## Introduction

Drainage in a chronic subdural hematoma (cSDH) is widely used and recommended, related to reduced recurrence and mortality rates [1]. It is not uncommon that cSDH presents with different densities in a mixed formation, with the variable acute solid component. The acute component is less likely to be drained and remains as a residual hematoma [2]. If the acute component is large enough, a craniotomy may be required to achieve adequate evacuation. Critically ill patients may not be stable enough to tolerate open surgeries or even transport to the operating room. If possible, less invasive treatments may be considered.

Bedside procedures for chronic subdural hematoma have been described [2,3]. The irrigation of the subdural space is usually more limited in this setting. In addition, the attempt to flush the subdural space can be dangerous, as the infused volume may further increase the intracranial pressure when associated with a valve mechanism (i.e., lack of fluid reflux). Moreover, proximal catheter occlusion can lead to additional procedures in up to 10% of patients [3]. For those reasons, the development of minimally invasive bedside therapies bears the potential of refining the armamentarium to treat selected cases of subdural hematoma (SDH).

## Case presentation

A 58-year-old male with a history of hypertension and alcohol abuse was found unresponsive at home after an overnight in a bed and was brought to the emergency room with a Glasgow Coma Scale (GCS) of three with fixed pupils, but remaining brainstem reflexes were present. No major signs of external trauma were observed on admission.

Brain computed tomography (CT) scan revealed a large left-sided hemispheric acute on chronic subdural hemorrhage (acSDH). There was a significant mass effect with 32mm hematoma width, 26mm midline shift (MLS), and subfalcine herniation. Blood tests revealed coagulopathy, hypokalemia, metabolic acidosis, hypoglycemia, abnormal liver function tests, thrombocytopenia, anemia, and elevated ethanol level (Table 1). Due to his critical neurologic condition, the patient was taken to the operation room (OR). 

**Table 1:** Laboratory data INR: international normalized ratio; APTT: activated partial thromboplastin time; pH: potential of hydrogen; Hb: hemoglobin; WBC: white blood cells.

	Admittance	1^st^ Post-Op	3^rd^ Post-Op	5^th^ Post-Op	10^th^ Post-Op
Prothrombin time (s)	18.1	18.0	18.8	17.6	16.1
INR	1.52	1.50	1.58	1.46	1.31
APTT (s)	32	34	33	32	31
Hb (g/dL)	8.4	8.3	8.9	9.3	9.2
Platelet (x10^3^/mcL)	95	124	101	133	189
Na/K (mmol/L)	138/3.3	149/3.7	158/3.2	152/3.2	137/3.7
Glucose (mg/dL)	51	135	119	104	78
pH	7.34	7.47	7.44	7.49	7.43
WBC (x10^3^/mcL)	7.3	11.0	6.5	12.7	12.5
Ethanol (mg/dL)	220 (nl 0-9)	NA	NA	NA	NA
Bilirubin (mg/dL)	1.6	2.7	2.3	1.4	2.4

In the OR, the patient became hemodynamically unstable. Hence, we decided to stop after doing the burr holes before converting them into a craniotomy flap to minimize the OR time. In addition, the bone flap removal with a sudden ICP drop could lead to further deterioration and asystole. Two left-sided burr holes were done at the point of maximal curvature, being one frontal and another parietal. The patient showed intraoperative signs of increased bleeding time. Fresh frozen plasma and platelets were administered during the procedure. An anti-thrombotic catheter originally FDA approved for intraventricular hemorrhage was used as subdural drainage with the intent of reducing postoperative obstruction (Codman® CerebroFlo® Endexo® from Integra LifeSciences, Princeton, NJ, USA), placed on the parietal burr hole and directed toward the frontal region.

In the immediate postoperative period, the neurologic exam improved to a GCS of six, and the CT scan revealed a residual MLS of 12.7mm due to the remaining acute component. As the patient was still in poor clinical condition, recombinant tissue-type plasminogen (r-tPA) solution was locally used to dissolve the acute component of the subdural hematoma. Recombinant tissue-type plasminogen activator was administered through the subdural catheter for two days at a dose of 1mg/mL to 2mL, followed by two-hour clamping. The administration was repeated every 12 hours for a total of four times. The catheter continued to drain for 10 days (Table 2). Once the radiologic result was good, we decided to remove the catheter on the 10^th^ day, without neurologic deterioration. 

**Table 2:** Drainage volume

Days	1	2	3	4	5	6	7	8	9	10
Drainage (mL)	20	32	18	287	278	346	338	291	244	140

**Figure 1:**
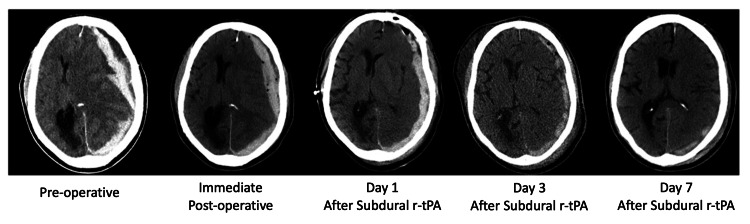
Computed tomography scans Computed tomography scans that show progressive radiologic improvement of the subdural hematoma. The subdural drain was removed on postoperative day 10.

Subsequent CT scans showed progressive resolution of the hematoma (Figure 1). No new hemorrhages or surgical-related infections occurred. The patient continued to recover and started to localize stimuli and intermittently follow simple commands around six weeks later. This final neurologic status was GCS 10T, with a tracheostomy, with an extended Glasgow Outcome Score of four. 

## Discussion

Twist drill bedside evacuation for cSDH may have similar rates of cure, recurrence, morbidity, and mortality rates when compared to burr holes done in the operating room [4]. The acute component of an acSDH is typically more solid, hence not suitable for catheter drainage as it won’t flow out. In addition to its consistency, its fragments contribute to clogging the catheter leading to proximal drain obstruction. In neurologically stable patients, we routinely delay the hematoma evacuation until it becomes more fluid to allow burr hole drainage. Nevertheless, if the acute component is predominant and the patient is neurologically unstable, emergent craniotomy is often required.

In our service, we routinely monitor clinical patients waiting for the acute SDH component to become more chronic and suitable for drainage. It’s important to mention that the residual acute component in the presented case was still large enough to sustain a severe midline shift. In addition, we typically see the mass effect increase during the period as it becomes more chronic, given the blood degradation leading to increased fluid accumulation. For patients with a large acute subdural component that cannot undergo open surgery, a prolonged subdural drain might be useful, albeit with a theoretical increased risk of infection and drain obstruction. 

In the presented case, the neurosurgical team opted for an anti-thrombotic catheter with a coated internal material that diminishes blood clot obstruction. The CerebroFlo® catheter (Codman® CerebroFlo® Endexo® from Integra LifeSciences, Princeton, NJ, USA) was originally developed to drain intraventricular hemorrhages, built with a large gauge and covered by a technology called Endexo®, according to the manufacturer, promotes a reduction of 99% of thrombus accumulation in vitro. It is described as a permanent additive that functionalizes all surfaces, including cut perforations, that greatly reduces fibrinogen and platelet activation and adhesion, resulting in less thrombus formation.

Limited reports of the subdural application of thrombolytic agents are available in the medical literature [2,5-8]. Based on previous publications that used local therapies with thrombolytic agents in cSDH, it was decided to use this approach to dissolve the subdural hematoma with r-tPA delivered via the catheter. The protocol used was the same described by Brazdzionis [8]. Two milliliters of R-tPA solution 1mg/mL followed by 2ml of saline flush were administered in the subdural space with the catheter being left clamped for two hours. It was repeated twice a day for a total of four times when it was not more necessary as the catheter continued to drain with no obstruction for another week. 

The goal of local thrombolytic therapy was to reduce the mass effect of the subdural hematoma, which is typically more difficult when larger acute components are present [2]. We hypothesize that the intrinsic catheter properties coupled with the thrombolytic agent played a decisive role in the success of this case. For the first time in the literature, this technique is reported, which yielded a meaningful recovery in this case that presented with a catastrophic condition. For this reason, we believe it is reasonable exploring further applications of this minimal strategy in the treatment of subdural hematoma. Although the catheter was still draining on the 10^th^ day, the aspect of the drainage was serous, and the CT improvement was very satisfactory, so it was decided to remove it, and the patient didn’t present neurologic deterioration after removal. 

Chronic SDH is a condition that presents an increasing incidence, including in developed countries, as the life expectancy continues to expand. The natural history of chronification of acute subdural hematomas takes about three weeks to occur. This patient had several factors related to delayed resorption, like high mid-line deviation, high hematoma thickness, coagulopathy, and thrombocytopenia [9]. The development of more refined bedside procedures to treat this condition could represent a valuable advance to minimize operative and anesthetic risks. 

Other authors also reported the use of subdural r-tPA application for acSDH, using traditional catheters, with good results, considering that r-tPA has more specificity to fibrin compared to Urokinase [10]. The anti-thrombotic catheter can improve the proximal risk of obstruction by clots, being a feasible alternative to the traditional catheter. A few of the disadvantages are that due to a larger gauge, it is more rigid than silicon and requires to be implanted cautiously, and its cost can be higher on a first simple analysis (estimated catheter cost is 30% higher than a traditional one). More studies with patients submitted to this type of drainage are required to compare if the results justify its use as a routine. 

## Conclusions

We presented a case in which subdural local therapy was delivered via an anti-thrombotic catheter. It was effective in treating the large acute component of a chronic subdural hematoma in a patient with poor neurologic and systemic conditions. As this is a case report, further studies are required to verify reproducibility and better understand the subset of patients that could benefit from this approach. This could, however, represent a viable option for the management of critically ill patients who would not tolerate craniotomy for a subdural hematoma. In addition, it could be beneficial to explore the bedside use of the anti-thrombotic catheter in acSDH patients with a less pronounced acute component without the use of r-tPA.
